# Correction: Investigating the causal effects of COVID-19 vaccination on the adoption of protective behaviors in Japan: Insights from a fuzzy regression discontinuity design

**DOI:** 10.1371/journal.pone.0342590

**Published:** 2026-02-10

**Authors:** Fengming Chen, Hayato Nakanishi, Yoichi Sekizawa, Sae Ochi, Mirai So

In Table 1, The titles of Columns 3 and 4 were transposed. The third column should be Born from April 1962 to March 1967 (n = 1,603) and the fourth column should be Born from April 1957 to March 1962 (n = 1,365). Please see the correct Table 1 here.

In Fig 1, the x-axis categories were not in the intended chronological order. Please see the correct Fig 1 here.

In S2 Table, the titles of Columns 3 and 4 were transposed. The third column should be Born from April 1962 to March 1967 (n = 1,603) and the fourth column should be Born from April 1957 to March 1962 (n = 1,365). In addition, for most rows (all except the “Age and Employed” rows), the data in Columns 4 and 5 were inadvertently swapped. Please view the correct S2 Table below.

**Table 1:** Characteristics of Study Participants in the Fourth Round of the Survey.

		Born from April 1962 to March 1967 (n = 1,603)	Born from April 1957 to March 1962 (n = 1,365)	Total (N = 12,067)
**Age, years**		61.7 (1.4)	66.6 (1.5)	53.6 (14.1)
**Gender**	Men	848 (52.9%)	715 (52.4%)	6,186 (51.3%)
	Women	755 (47.1%)	650 (47.6%)	5,881 (48.7%)
**Highest education level, No. (%)**	Junior/senior high school	435 (27.1%)	420 (30.8%)	3,728 (30.9%)
	Two- or three-year college	361 (22.5%)	277 (20.3%)	2,594 (21.5%)
	Four-year college or higher	807 (50.3%)	668 (48.9%)	5,745 (47.6%)
**Marital status, No.**	Married	1,178 (73.5%)	1,053 (77.1%)	7,617 (63.1%)
**(%)**	Divorced	131 (8.2%)	102 (7.5%)	721 (6.0%)
	Bereaved	50 (3.1%)	66 (4.8%)	305 (2.5%)
	Never married	244 (15.2%)	144 (10.5%)	3,424 (28.4%)
**Employed, %**		62.3%	42.9%	60.9%
**Lifestyle, %**	Avoiding going to poorly ventilated places	90.1%	93.3%	88.9%
	Avoiding going to crowded places	91.1%	92.7%	90.1%
	Avoiding conversing or vocalizing near others	88.5%	89.7%	86.7%
	Wearing a mask	98.6%	98.3%	97.5%
	Handwashing	96.1%	97.6%	95.9%
	Sanitizing hands	90.8%	91.4%	90.9%
	Changing clothes frequently	26.4%	25.8%	28.2%
	Gargling	65.9%	69.5%	69.0%
	Sanitizing personal belongings	27.3%	26.7%	33.5%
	Keeping people at a distance when going out	87.5%	89.7%	85.7%
	Refraining from visiting medical facilities	43.7%	44.4%	47.9%
	Avoiding going outside	66.1%	70.8%	67.7%
**Frequency of going out, No. (%)**	Almost everyday	492 (30.7%)	328 (24.0%)	3,458 (28.7%)
	4–5 days per week	412 (25.7%)	373 (27.3%)	3,185 (26.4%)
	2–3 days per week	395 (24.6%)	394 (28.9%)	2,881 (23.9%)
	1 day per week	233 (14.5%)	203 (14.9%)	1,755 (14.5%)
	1 day per month	30 (1.9%)	37 (2.7%)	369 (3.1%)
	Not at all	41 (2.6%)	30 (2.2%)	419 (3.5%)
**Frequency of meeting acquaintances, No. (%)**	Almost everyday	80 (5.0%)	74 (5.4%)	738 (6.1%)
	A few times per week	241 (15.0%)	260 (19.0%)	1,792 (14.9%)
	Once per week	202 (12.6%)	250 (18.3%)	1,535 (12.7%)
	Once per two weeks	171 (10.7%)	137 (10.0%)	1,174 (9.7%)
	Once per month	280 (17.5%)	201 (14.7%)	1,992 (16.5%)
	Not at all	629 (39.2%)	443 (32.5%)	4,836 (40.1%)
**Frequency of vaccination, No. (%)**	0	827 (51.6%)	224 (16.4%)	6,768 (56.1%)
	1	597 (37.2%)	314 (23.0%)	2,413 (20.0%)
	2	179 (11.2%)	827 (60.6%)	2,886 (23.9%)

**Fig 1:**
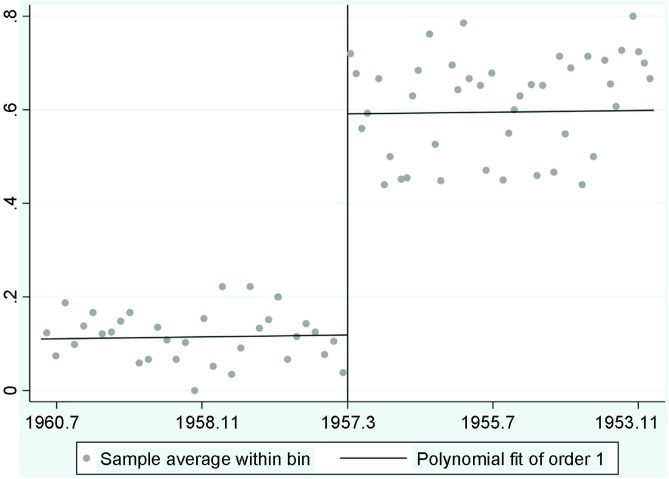
RD plot of the probability of receiving the vaccine twice.

## Supporting information

S2 Table Characteristics of Study Participants in the First Survey Round. (DOCX)

## References

[1] Chen F, Nakanishi H, Sekizawa Y, Ochi S, So M. Investigating the causal effects of COVID-19 vaccination on the adoption of protective behaviors in Japan: Insights from a fuzzy regression discontinuity design. PLoS One. 2024;19(6):e0305043. doi: 10.1371/journal.pone.0305043 38865314 PMC11168682

