# Supplementary material for: Investigating the causal effects of COVID-19 vaccination on the adoption of protective behaviors in Japan: Insights from a fuzzy regression discontinuity design
Source: PLoS One. 2024 Jun 12;19(6):e0305043. doi: 10.1371/journal.pone.0305043 (PMC11168682; doi:10.1371/journal.pone.0305043)
Supplement: S1 Table — (DOCX) [file pone.0305043.s002.docx]

**S1 Table. Definitions of Variables.**

| **Variable** | **Question** |
| --- | --- |
| **Binary variables (Yes = 1, No = 0)** | **Do you have any awareness about the following things to avoid getting infected with the novel coronavirus?** |
| 1. Avoiding going to poorly ventilated places | Avoid going to poorly ventilated places |
| 1. Avoiding going to crowded places | Avoid going to crowded places |
| 1. Avoiding conversing or vocalizing near others | Avoid close contact with others during conversations or speaking |
| 1. Wearing a mask | Wear a mask |
| 1. Handwashing | Wash hands |
| 1. Sanitizing hands | Sanitize hands |
| 1. Changing clothes frequently | Change clothes frequently |
| 1. Gargling | Gargle |
| 1. Sanitizing personal belongings | Sanitize personal belongings |
| 1. Keeping people at a distance when going out | Keep people at a distance when going out |
| 1. Refraining from visiting medical facilities | Refrain from visiting medical facilities |
| 1. Avoiding going outside | Avoid going outside |
| **Continuous Variables** | |
| 1. Frequency of going out | How often have you gone out in the past one month?  (reversed order)   1. Not at all 2. 1 day per month 3. 1 day per week 4. 2‒3 days per week 5. 4‒5 days per week 6. Almost every day |
| 1. Frequency of meeting acquaintances | How frequently have you been directly meeting acquaintances, such as relatives, friends, and neighbors, excluding those living with you and work-related interactions, in the past month? (reversed order)   1. Not at all 2. Once per month 3. Once per two weeks 4. Once per week 5. A few times per week 6. Almost every day |
