# Supplementary material for: Investigating the causal effects of COVID-19 vaccination on the adoption of protective behaviors in Japan: Insights from a fuzzy regression discontinuity design
Source: PLoS One. 2024 Jun 12;19(6):e0305043. doi: 10.1371/journal.pone.0305043 (PMC11168682; doi:10.1371/journal.pone.0305043)
Supplement: S3 Table — (DOCX) [file pone.0305043.s004.docx]

**S3 Table. Estimation Result of the Effect of Eligibility on Vaccination Rates (Treatment: Twice).**

| **Outcome variable** | **Point estimate** | **95% CI** | ***p*-value** | **Bandwidth (months)** | **Total sample** | **Analyzed samples** | |
| --- | --- | --- | --- | --- | --- | --- | --- |
|  |  |  |  |  |  | **Control** | **Treatment** |
| **Vaccinated twice** | 0.473 | (0.394–0.572) | < 0.001 | 42.149 | 12,067 | 1,191 | 1,024 |

CI, confidence interval. The outcome variable is binary, being one if vaccinated twice, and zero otherwise.
