# Supplementary material for: Investigating the causal effects of COVID-19 vaccination on the adoption of protective behaviors in Japan: Insights from a fuzzy regression discontinuity design
Source: PLoS One. 2024 Jun 12;19(6):e0305043. doi: 10.1371/journal.pone.0305043 (PMC11168682; doi:10.1371/journal.pone.0305043)
Supplement: S4 Table — (DOCX) [file pone.0305043.s005.docx]

**S4 Table. Estimation Results of the Effect of Eligibility on Vaccination Rates (Treatment: At Least Once).**

| **Outcome variable** | **Point estimate** | **95% CI** | ***p*-value** | **Bandwidth (months)** | **Total sample** | **Analyzed samples** | |
| --- | --- | --- | --- | --- | --- | --- | --- |
|  |  |  |  |  |  | **Control** | **Intervention** |
| **Vaccinated at least once** | 0.326 | (0.236 to 0.444) | <0.001 | 39.298 | 8,814 | 1,091 | 954 |

CI, confidence interval. The outcome variable is binary, being one if vaccinated at least once, and zero otherwise.
