# Supplementary material for: Investigating the causal effects of COVID-19 vaccination on the adoption of protective behaviors in Japan: Insights from a fuzzy regression discontinuity design
Source: PLoS One. 2024 Jun 12;19(6):e0305043. doi: 10.1371/journal.pone.0305043 (PMC11168682; doi:10.1371/journal.pone.0305043)
Supplement: S5 Table — (DOCX) [file pone.0305043.s006.docx]

**S5 Table. Estimation Results of the Regression Discontinuity Design on Outcomes (Treatment: At Least Once).**

| **Outcome variables** | **Point estimate** | **95% CI** | ***p*-value** | **Bandwidth (months)** | **Total sample** | **Analyzed samples** | |
| --- | --- | --- | --- | --- | --- | --- | --- |
|  |  |  |  |  |  | **Control** | **Intervention** |
| **Wearing a mask** | 0.00 | (-0.10–0.13) | 0.795 | 45.40 | 12,067 | 1,267 | 1,072 |
| **Handwashing** | 0.05 | (-0.08–0.20) | 0.409 | 43.34 | 12,067 | 1,215 | 1,042 |
| **Avoiding going outside** | 0.04 | (-0.29–0.34) | 0.882 | 41.45 | 12,067 | 1,160 | 1,003 |
| **Avoiding going to poorly ventilated places** | 0.06 | (-0.14–0.28) | 0.516 | 41.76 | 12,067 | 1,160 | 1,003 |
| **Avoiding going to crowded places** | -0.07 | (-0.26–0.12) | 0.461 | 46.73 | 12,067 | 1,287 | 1,084 |
| **Avoiding conversing or vocalizing near others** | 0.04 | (-0.19–0.27) | 0.750 | 40.04 | 12,067 | 1,118 | 983 |
| **Sanitizing hands** | 0.08 | (-0.10–0.31) | 0.327 | 47.07 | 12,067 | 1,297 | 1,102 |
| **Changing clothes frequently** | -0.24 | (-0.59–0.02) | 0.069 | 38.82 | 12,067 | 868 | 809 |
| **Gargling** | -0.10 | (-0.43–0.19) | 0.447 | 47.30 | 12,067 | 1,297 | 1,102 |
| **Sanitizing personal belongings** | 0.09 | (-0.17–0.42) | 0.400 | 53.84 | 12,067 | 1,444 | 1,224 |
| **Keeping people at a distance when going out** | 0.04 | (-0.16–0.25) | 0.695 | 52.16 | 12,067 | 1,418 | 1,209 |
| **Refraining from visiting medical facilities** | 0.14 | (-0.20–0.47) | 0.426 | 54.30 | 12,067 | 1,465 | 1,242 |
| **Frequency of going out** | -0.69 | (-1.56–0.15) | 0.105 | 42.53 | 12,067 | 1,191 | 1,024 |
| **Frequency of meeting acquaintances** | 0.10 | (-1.27–1.35) | 0.956 | 29.49 | 12,067 | 775 | 724 |

CI: confidence interval.
