# Supplementary material for: Investigating the causal effects of COVID-19 vaccination on the adoption of protective behaviors in Japan: Insights from a fuzzy regression discontinuity design
Source: PLoS One. 2024 Jun 12;19(6):e0305043. doi: 10.1371/journal.pone.0305043 (PMC11168682; doi:10.1371/journal.pone.0305043)
Supplement: S6 Table — (DOCX) [file pone.0305043.s007.docx]

**S6 Table. Estimation Results of the Regression Discontinuity Design on Outcomes in the First Survey Round.**

| **Outcome variables** | **Point estimate** | **95% CI** | ***p*-value** | **Bandwidth (months)** | **Total sample** | **Analyzed samples** | |
| --- | --- | --- | --- | --- | --- | --- | --- |
|  |  |  |  |  |  | **Control** | **Intervention** |
| **Wearing a mask** | 0.01 | (-0.03–0.05) | 0.618 | 104.36 | 12,067 | 2,658 | 2,575 |
| **Handwashing** | -0.00 | (-0.05–0.04) | 0.851 | 59.16 | 12,067 | 1,583 | 1,355 |
| **Avoiding going outside** | 0.15 | (-0.05–0.37) | 0.144 | 51.78 | 12,067 | 1,392 | 1,191 |
| **Avoiding going to poorly ventilated places** | 0.05 | (-0.07–0.19) | 0.363 | 50.99 | 12,067 | 1,370 | 1,168 |
| **Avoiding going to crowded places** | 0.02 | (-0.07–0.13) | 0.573 | 95.81 | 12,067 | 2,434 | 2,281 |
| **Avoiding conversing or vocalizing near others** | 0.04 | (-0.08–0.23) | 0.329 | 55.47 | 12,067 | 1,483 | 1,269 |
| **Sanitizing hands** | 0.03 | (-0.08–0.15) | 0.542 | 58.91 | 12,067 | 1,558 | 1,339 |
| **Changing clothes frequently** | -0.10 | (-0.30–0.07) | 0.211 | 44.81 | 12,067 | 1,232 | 1,061 |
| **Gargling** | -0.02 | (-0.24–0.17) | 0.758 | 52.68 | 12,067 | 1,418 | 1,209 |
| **Sanitizing personal belongings** | 0.11 | (-0.08–0.30) | 0.245 | 51.84 | 12,067 | 1,392 | 1,191 |
| **Keeping people at a distance when going out** | -0.02 | (-0.16–0.16) | 0.967 | 45.91 | 12,067 | 1,267 | 1,072 |
| **Refraining from visiting medical facilities** | 0.12 | (-0.05–0.32) | 0.157 | 76.18 | 12,067 | 1,978 | 1,720 |
| **Frequency of going out** | -0.06 | (-0.50–0.60) | 0.847 | 55.63 | 12,067 | 1,483 | 1,269 |
| **Frequency of meeting acquaintances** | 0.26 | (-0.37–0.77) | 0.490 | 88.37 | 12,067 | 2,249 | 2,083 |

CI: confidence interval. The outcome variables of frequency of going out and frequency of meeting acquaintances are continuous, ranging from 1 (not at all) to 6 (almost every day). Other outcome variables are binary, with one for conducting the behavior and zero otherwise.
