# Supplementary material for: Investigating the causal effects of COVID-19 vaccination on the adoption of protective behaviors in Japan: Insights from a fuzzy regression discontinuity design
Source: PLoS One. 2024 Jun 12;19(6):e0305043. doi: 10.1371/journal.pone.0305043 (PMC11168682; doi:10.1371/journal.pone.0305043)
Supplement: Attachment — Submitted filename: Response_to_Reviewers.docx [file pone.0305043.s008.docx]

**AUTHORS’ RESPONSES TO REVIEWER #1’S COMMENTS**

We would like to express our sincere gratitude to both the reviewers and the editor for their extremely helpful and constructive comments on our manuscript, which have helped improve it significantly. We have done our best to address every point raised by the reviewers.

**Reviewer #1**:

The manuscript seeks to understand the relationship between vaccination and protective behaviors against Covid-19. It uses an interesting, little-used methodology that deserves to be highlighted. Few changes are suggested in the text.
**(Comment 1)** Pag. 3, line 53 (Introduction): Correct this sentence. Vaccination does not prevent infection in anyone, it only improves the individual's immune response.

**Response 1:** Thank you for the reviewer’s comment. We have revised this inaccurate statement and replaced the reference as follows.

**Pag.4, lines 73-80(Manuscript_change track):**

”While vaccination decreases the likelihood of hospitalization and may mitigate COVID-19 symptoms, it is not an infallible solution to the issue of COVID-19. This is because, following vaccination, some individuals continue to exhibit weak immune responses[1]. Hence, there is a concern that people who believe a lower risk of being infected or experiencing complications through vaccination take fewer protective measures, which may paradoxically lead to the spread of the virus [2–3].”

1.Lipsitch M, Krammer F, Regev-Yochay G, Lustig Y, Balicer RD.  SARS-CoV-2 breakthrough infections in vaccinated individuals: Measurement, causes and impact. Nat Rev Immunol. 2022;22:57–65. doi:10.1038/s41577-021-00662-4

**Response 2**: We conducted a thorough review of the reference list and main text, implementing minor revisions focused mainly on the inclusion of DOIs and the correction of inaccurate statements. Previously omitted, DOIs have now been incorporated into the revised manuscript.

**Reviewer #2**:

The paper utilizes RDD (Regression Discontinuity Design) to estimate the impact of COVID-19 vaccination on the protective behaviors of individuals in Japan. In this review, I aim to enhance the article's quality by addressing some critical issues.

**(Comment 1)**Regarding the abstract, I suggest the following improvements:
a) Clarity and Brevity: simplify the abstract by focusing on the primary findings and the study's significance. This could involve shortening sentences and eliminating non-essential details for improved clarity and readability. For example, there's no need to repeat "quasi-experimental design" twice in the same section (background).
b) Emphasize Key Results: clearly articulate the main study findings in the abstract, especially those related to the influence of COVID-19 vaccination on protective behaviors. This will provide readers with a quick grasp of the study's outcomes. The authors have adequately reported the main results, but I believe it would be more informative to reference other studies on the topic and explain how the results confirm or contradict existing evidence on the subject.
c) Implications and Context: briefly touch upon the broader implications of the findings and their contributions to existing knowledge or public health policies. This will help readers understand the research's significance within the broader context of managing the COVID-19 pandemic.

**Response 1:** Thank you for the reviewer’s extensive suggestions on the abstract. Following the advice, we rewrote the abstract as follows.

**Pag.2-3, lines, 21-63(Manuscript_change track):**

“**Abstract**

**Background**: During the COVID-19 pandemic, concerns emerged that vaccinated individuals might engage less in infection-preventive behaviors, potentially contributing to virus transmission. This study evaluates the causal effects of COVID-19 vaccination on such behaviors within Japan, highlighting the significance of understanding behavioral dynamics in public health strategies.

**Methods**: Utilizing Japan's age-based vaccination priority for those born before April 1, 1957, this research employs a regression discontinuity design (RDD) to assess the vaccination's impact. Data from the fourth round of a longitudinal online survey, conducted by the Research Institute of Economy, Trade, and Industry from July 20 to 27, 2021, served as the basis for analyzing 14 infection protective behaviors, including mask usage, handwashing, and avoiding crowds.

**Results:** A total of 12067 participants completed the survey. The analyzed sample size varied by outcome variable, ranging from 1499 to 5233. Analysis revealed no significant differences in the 14 behaviors examined among fully vaccinated, partially vaccinated, and unvaccinated individuals. This consistency across groups suggests that vaccination status did not significantly alter engagement in protective behaviors during the observation period.

**Conclusions:** Empirical findings highlight the complexity of behavioral responses following vaccination, indicating that such responses may be influenced by various factors, rather than vaccination status alone. Additionally, this result underscores the importance of crafting public health policies that account for the intricate interplay between vaccination and behavior. This study contributes to the broader discourse on managing responses to the pandemic and tailoring interventions to sustain or enhance protective health behaviors amid vaccination rollouts.”

**(Comment 2)**Regarding data accessibility, I am interested in whether the data and code are publicly available. Transparency is a fundamental aspect of scientific research, and there is no reason to keep this data confidential. It's crucial to emphasize that my acceptance of the paper is contingent on the availability of data and code in a public repository, such as GitHub or Dataverse.
Upon further examination of the submission, I came across the statement: "Data cannot be publicly shared because it is owned by the RIETI, and the authors lack permission to do so. The data can be accessed through the RIETI by researchers who meet the criteria for accessing confidential data."
Unfortunately, due to this data limitation, I must recommend rejecting the paper. PLOS ONE is a highly prestigious journal, and I believe that a focus on open data policies will further enhance its prestige. Without accessible data, it becomes impossible to verify the reliability of the results. If we cannot scrutinize the data, we cannot challenge or validate your findings, which goes against the principles of scientific inquiry.

**Response 2:** As a response to the reviewer’s concern regarding data availability, which is a critical aspect of the peer review process and necessary for replication studies, we acknowledge the importance of sharing our data.

The dataset in question is owned by the Research Institute of Economy, Trade, and Industry (RIETI). We have consulted with RIETI officers responsible for data management, and they have not approved the disclosure of the data for the following reasons:

1. RIETI does not have the policy and guidelines to make the data public.

2. Although the dataset is anonymous, certain combinations of variables, such as birth year and month, and postal code of residence, may lead to the identification of personal information. Revealing personal information from the dataset would violate the agreement with the study participants and the Japanese Personal Information Protection Law.

Therefore, RIETI officers must be cautious about making the data public. However, while the data are not freely available to everyone, researchers who meet the criteria for accessing confidential data can obtain access through RIETI(contact via e-mail: info@rieti.go.jp). This requires approval from the ethical committee of the hospital, a commitment to strict data management for confidentiality protection, and other procedural commitments. We are prepared to assist researchers interested in accessing the data from RIETI.

Additionally, we have made the Stata codes available via protocols.io as follows:doi:[dx.doi.org/10.17504/protocols.io.yxmvm38pnl3p/v1](https://dx.doi.org/10.17504/protocols.io.yxmvm38pnl3p/v1)

**Response 3**: We conducted a thorough review of the reference list and main text, implementing minor revisions focused mainly on the inclusion of DOIs and the correction of inaccurate statements. Previously omitted, DOIs have now been incorporated into the revised manuscript.
