# Supplementary material for: Investigating the causal effects of COVID-19 vaccination on the adoption of protective behaviors in Japan: Insights from a fuzzy regression discontinuity design
Source: PLoS One. 2024 Jun 12;19(6):e0305043. doi: 10.1371/journal.pone.0305043 (PMC11168682; doi:10.1371/journal.pone.0305043)
Supplement: Attachment — Submitted filename: Response_to_Reviewers.docx [file pone.0305043.s009.docx]

**AUTHORS’ RESPONSES TO THE EDITOR**

We would like to express our sincere gratitude to the editor for their insightful and constructive feedback on our manuscript. Following the suggestions, we have carefully revised the manuscript and addressed each comment in detail. We provide a point-by-point response to issues raised as follows.

**(Comment 1)** I still understand that there are questions about the replicability of the study proposed here. I would ask you to incorporate into the text of the manuscript information relevant to obtaining the data, following the recommendations of the Plos One Data Availability Policy.

**Response 1:** We appreciate this comment and have updated the manuscript accordingly. Specifically, on **page 7, lines 145–154** (Manuscript_change track), we have included detailed information about how the dataset, managed by RIETI, is not publicly accessible due to legal and policy constraints. However, RIETI permits data access to researchers who meet strict confidentiality criteria. Full details and contact information for data access requests are now clearly stated in the manuscript.

**(Comment 2)** I would also be interested in some clarification on the sentence "We requested that all eligible respondents from the first round of the survey participate in the subsequent survey rounds via email" on page 06 of the revised version of the manuscript. The sentence indicates that the authors were part of the data collection process. If this was the case, it is no longer a third part dataset, as was first understood.

**Response 2:** Thank you for highlighting this need for clarity. To address this, we have revised the manuscript **pages 6–7, lines 117–138** (Manuscript_change track) to clarify that although YS, a co-author, was involved in designing the survey and managing the data collection process as an employee of RIETI, the actual data ownership and rights to distribute remain with RIETI. This makes the dataset “third-party data” under PLOS ONE’s definitions, as the authors do not own the dataset nor have the rights to freely distribute it. Detailed explanations of the author roles and processes have been added to ensure transparency.

**Editorial Request**: Please ensure that your Response to Reviewers letter includes a point-by-point response to all comments provided by the editor and reviewers.

**Response 3:** We have reviewed the entire manuscript and reference list, making the necessary revisions including the addition of two new references and corrections to inaccuracies pointed out during the review. Each point raised by the reviewers has been addressed as shown in this letter, and we believe the revisions have greatly improved the manuscript.
