# Supplementary material for: Correction: Investigating the causal effects of COVID-19 vaccination on the adoption of protective behaviors in Japan: Insights from a fuzzy regression discontinuity design
Source: PLoS One. 2026 Feb 10;21(2):e0342590. doi: 10.1371/journal.pone.0342590 (PMC12890088; doi:10.1371/journal.pone.0342590)
Supplement: S2 Table — (DOCX) [file pone.0342590.s001.docx]

**S2 Table. Characteristics of Study Participants in the First Survey Round.**

|  |  | **Born from April 1962 to March 1967**  **(n = 1,987)** | **Born from April 1957 to March 1962 (n = 1,656)** | **Total**  **(N = 16,642)** |
| --- | --- | --- | --- | --- |
| **Age, years** |  | 61.0 (1.4) | 65.8 (1.5) | 49.8 (15.7) |
| **Gender** | Men | 979 (49.3%) | 822 (49.6%) | 8,022 (48.2%) |
|  | Women | 1,008 (50.7%) | 834 (50.4%) | 8,620 (51.8%) |
| **Highest** | Junior/senior high school | 574 (28.9%) | 520 (31.4%) | 5,530 (33.2%) |
| **education** | Two- or three-year college | 461 (23.2%) | 334 (20.2%) | 3,502 (21.0%) |
| **level, No. (%)** | Four-year college or higher | 952 (47.9%) | 802 (48.4%) | 7,610 (45.7%) |
| **Marital status,** | Married | 1,462 (73.6%) | 1,284 (77.5%) | 9,905 (59.5%) |
| **No. (%)** | Divorced | 171 (8.6%) | 131 (7.9%) | 949 (5.7%) |
|  | Bereaved | 61 (3.1%) | 79 (4.8%) | 366 (2.2%) |
|  | Never married | 293 (14.7%) | 162 (9.8%) | 5,422 (32.6%) |
| **Employed, %** |  | 63.3% | 45.4% | 60.4% |
| **Lifestyle, %** | Avoiding going to poorly ventilated places | 88.5% | 91.4% | 85.1% |
|  | Avoiding going to crowded places | 88.3% | 89.9% | 86.7% |
|  | Avoiding conversing or vocalizing near others | 84.8% | 84.7% | 79.7% |
|  | Wearing a mask | 97.7% | 98.3% | 97.1% |
|  | Handwashing | 97.3% | 97.9% | 96.3% |
|  | Sanitizing hands | 90.2% | 91.2% | 89.5% |
|  | Changing clothes frequently | 20.9% | 20.4% | 21.5% |
|  | Gargling | 68.0% | 69.4% | 68.7% |
|  | Sanitizing personal belongings | 25.3% | 25.9% | 29.2% |
|  | Keeping people at a distance when going out | 84.5% | 86.7% | 81.9% |
|  | Refraining from visiting medical facilities | 47.6% | 47.6% | 50.2% |
|  | Avoiding going outside | 58.9% | 65.2% | 61.0% |
| **Frequency of** | Almost everyday | 561 (28.2%) | 388 (23.4%) | 4,373 (26.3%) |
| **going out, No.** | 4–5 days per week | 492 (24.8%) | 424 (25.6%) | 4,087 (24.6%) |
| **(%)** | 2–3 days per week | 534 (26.9%) | 484 (29.2%) | 4,261 (25.6%) |
|  | 1 day per week | 317 (16.0%) | 260 (15.7%) | 2,836 (17.0%) |
|  | 1 day per month | 51 (2.6%) | 52 (3.1%) | 642 (3.9%) |
|  | Not at all | 32 (1.6%) | 48 (2.9%) | 443 (2.7%) |
| **Frequency of** | Almost everyday | 104 (5.2%) | 98 (5.9%) | 1,055 (6.3%) |
| **meeting** | A few times per week | 320 (16.1%) | 356 (21.5%) | 2,588 (15.6%) |
| **acquaintances,** | Once per week | 297 (14.9%) | 315 (19.0%) | 2,454 (14.7%) |
| **No. (%)** | Once per two weeks | 203 (10.2%) | 179 (10.8%) | 1,858 (11.2%) |
|  | Once per month | 419 (21.1%) | 283 (17.1%) | 3,279 (19.7%) |
|  | Not at all | 644 (32.4%) | 425 (25.7%) | 5,408 (32.5%) |
